# Comparing achievements of medical graduates in an alternative unique pre-medical track vs regular medical track

**DOI:** 10.5116/ijme.62f6.10b6

**Published:** 2022-09-02

**Authors:** Shimon Amar, Elena Chernin, Gabriel Schreiber, Michael Friger, Angel Porgador

**Affiliations:** 1 Faculty of Health Sciences, Ben-Gurion University of the Negev, Beer-Sheva, Israel

**Keywords:** Premed/medical students, six-year program, final exam grades

## Abstract

**Objectives:**

To evaluate the association between the achievements of medical students and whether they were admitted via the pre-medical track or the regular direct track.

**Methods:**

We performed a comparative retrospective data study using data from a three-year experimental cohort in a six-year medical school. We analyzed the academic achievements of all students admitted at one Israeli medical school between 2013-2015, either directly to the six-year program or via a pre-medical track. We compared averages of both yearly final grades and final medical examinations grades between the two groups. Descriptive statistics were calculated and differences between groups were evaluated using multivariate analysis.

**Results:**

Of the 324 students included in the study, 65 (20.1%) were enrolled in all three cohorts of the pre-medical track. Age and Gender distribution were nearly similar for both tracks. For the first two cohorts, the average final grades of year one of pre-medical students were significantly higher than those of regular direct track (F=_(3,167) _6.10, p=0.001), but the opposite was true for the third cohort (F=_(3,110)_2.38, p=0.073). No further statistically significant differences were found neither between the groups in their final exams grades nor between choosing a MD/PhD optional track and admission pathway.

**Conclusions:**

Our results suggest promising achievements with the pre-medical admission pathway. This should encourage further discussion about the significant potential human resources lost by current admission processes and may question the effectiveness of six-year programs in medical schools.

## Introduction

Every year, medical schools throughout the world are tasked with selecting a handful of candidates out of many eligible applicants. The decisions made in the selection process have implications for public health.^1^ Thus, apart from cognitive ability, selection criteria include character attributes, such as altruism, empathy, reliability, communication skills, and others.^2^ Validating the effectiveness of the selection process is highly complex and requires a lengthy follow-up during the integration of graduates into the medical workforce and their subsequent years in practice.^3^ Attempts to validate the admission process are presented in a large body of literature that focuses on students' achievements in admission tests, through their years of study, and in their final examinations. The studies provide substantial evidence for a direct correlation between admission scores and academic achievements in the following years.^4^^-^^7^ In contrast, evidence for the reliability of interviews and other humanistic models is limited,^8^ and the ability of these models to examine personal qualities or predict academic achievements and clinical performance is not well established.^9^

Integrating pre-medical studies is a way to deal with medical school admission process limitations. A pre-medical program, common in the United States and Canada, is an educational, academic track that qualifies students and facilitates their subsequent selection into medical school. The pre-medical track offers courses on core subjects that prepare students for medical studies. These include courses for allied health professions, community involvement, clinical experience, and research experience. Some pre-medical programs provide broad-based preparation for professional tracks and can prepare students for entry into a variety of primary professional programs or graduate degrees with similar prerequisites (including schools of medicine, veterinary medicine, and pharmacy.) Most studies show a positive correlation between student achievement in pre-medical programs and their achievements through their years in medical school.^10^

Yet, pre-medical programs are not offered by the majority of universities and colleges across the world. Many medical schools consider high school studies as 'pre-medicine'. A student in an academic pre-medical track can choose an undergraduate program in any field, as long as prerequisite courses for the medical program are included. The courses primarily focus on the scientific fields of biology, chemistry, organic chemistry, brain sciences, behavioral sciences, and physics, which provide the necessary preparation for the MCAT entrance exam and meet the admission requirements of medical schools.^11^ For this reason, students in the pre-medical track, tend to choose a major related to one of these scientific fields. However, in recent years, a growing number of students with a background in the humanities are applying for medical school, a trend that is well-received by the schools. For example, the Mount Sinai School of Medicine established a specific program for non-scientific majors. The Humanities and Medicine Program (HuMed) caters to graduates in humanities and social sciences and does not require the MCAT or scientific courses.^12^

At Ben Gurion University, medical studies have been taught for 49 years as a six-year program. Admission to medical school is based on final school grades, an interview, and a computerized personality test, or alternatively, on two interviews (MED track). The number of applicants increases every year, and it is five times higher than the admission quotas. The same is true of the other three established medical schools in Israel. In 2013, for the first time at our school, we trialed the "Graduate in Medical Sciences" study program as a three-year program that provided a pre-medicine track (PREMED track). The program was intended for candidates who achieved high scores in the admission process, including the interviews but were not among those admitted to the medical school because of the limited number of places.

This unique PREMED program was designed from its outset to provide an alternative track where students study basic sciences at an undergraduate level and acquire basic clinical knowledge in a program that is nearly identical to that of the six-year medical track. An additional focus of the program was a specifically designed biomedical research course. Graduates could continue directly to the fourth year of medical studies, either in the regular MD track or the MD/PhD clinician-researcher track. Other options for graduates included medical studies in a four-year program at a different academic institution or studying towards a Master's degree at Ben Gurion University or any other academic institution.

At first, it was suggested that only the top 60% of graduates who successfully completed the 'Graduate in Medical Sciences' PREMED program could continue their medical studies at the Joyce and Irving Goldman School of Medicine at the Ben-Gurion University of the Negev. However, in practice, based on considerations of the health system needs in Israel, admission quotas have increased annually. Therefore, the PREMED program was gradually shortened, and the students joined the six-year medical program. The first cohort of students attended the PREMED program for two years before they joined the third year of the six-year medical program, the second cohort joined the medical program after one year, and the third cohort joined the direct medical program right from the beginning.

At a time when the number of doctors and the quality of medical training appears to be out of balance, we wish to explore, through the opportunity provided by the three cohorts of the PREMED program, the potential achievements of students who were not initially admitted to medical school, some of whom would likely choose to study elsewhere in Israel or abroad. Alternatives to the traditional six-year program could provide worthy academic options that would allow for an increase in the number of locally trained students.

The objective of our study was to evaluate the association between the achievements of medical students and whether they were admitted via the PREMED track or the regular direct track.

## Methods

### Study design and participant

A retrospective study was conducted on three consecutive cycles of medical students between 2013 and 2015. The study was approved by the local Institutional Review Board (College of Human Sciences Research Ethics Committee) for using retrospective students' achievements records. Because of the retrospective observational design of the study and in accordance with Israeli law, written informed consent was not required. All data were analyzed anonymously by our exam department at the school of medicine.

### Data collection

A total of Three hundred and twenty-four students who enrolled in both MED and PREMED admission tracks to BGU medical school were included in this study. We compared the yearly final grades and the final medical examinations grades of all participants. The data collected included: gender, birth year, yearly grade means and medians, weighted decile values, and final examination grades. The records were then divided into two groups: those of students admitted via the PREMED track and those of students in the direct medical track, MED.

Inclusion criteria were all students admitted to the medical school between 2013 and 2015 either in the direct track or the PREMED track. Exclusion criteria were students admitted during these years who discontinued their studies in the clinical years and did not take the final examinations (a total of four students, all in the direct track.)

Of all students, 65 (20.1%) were in the PREMED track. Stratified by year, the PREMED students comprised 12.6% of all students admitted in 2013 (11/89), 16.8% (18/107) of students in the 2014 class, and 28.1% (36/128) in the 2015 class.

Data for the two cohorts of 2013 and 2014 were combined in the analysis tables because their study structure was similar to each other and different from that of students in the direct track. Students in the third cohort of 2015 joined the direct medical track in the first year, and their data were analyzed separately.

The analysis of the student's achievements is demonstrated by two different record components. The first component refers to the annual grade average and is based on 10-14 academic courses taught each academic year. The second component refers to the final exam scores. The Israeli equivalent of the USMLE or the UEMS exams consists of five separate professional exams: Internal medicine, Pediatrics, Gynecology, Surgery and Psychiatry.

### Statistical analysis

Statistical analysis was carried out in three stages. The first stage entailed descriptive statistics. Means, medians and standard deviations are presented for continuous variables and distribution and percentages for categorical variables. The second stage was univariate analysis. Outcome variables were compared between the groups and between other predictive variables, such as age and gender. We used a T-test to compare students' ages and a χ^2^ test to compare gender between the two groups. We compared students' yearly final grades and their grades in the final medical examinations. A T-test was used to compare the mean grades between the two groups. In the third stage, a multivariate analysis was carried out by linear regression and quantile regression (for quantiles: 10, 25, 50, 70, 90 and 95). The two methods were used to evaluate the interaction between study track and age and interaction between study track and gender. SPSS was used for data processing and analysis. Tests were 2-tailed, and statistical significance was set at p < 0.05.

## Results

### Demographics of the study cohorts

We compared the grades of 324 students divided into two groups. In the first and second cohorts, 29 (17%) and 167 (83%) students were admitted into the PREMED and direct MED programs, respectively. The third cohort comprised 36 (28%) and 92 (72%) students in the PREMED and the direct MED programs, respectively.

There was no significant difference in gender distribution between the two groups in any of the three cohorts. A comparison of students' age found that in the third cohort, students in the direct MED track were slightly older (M=30.9, SD=2.02) years than students in the PREMED track (M=30.1, SD=1.83) years, (t_(__113)_=2.009, p=0.049). The results are presented in Table 1.

### Average final grades comparison

Our comparison of the final grades between the MED and PREMED groups in the three cohorts focused on the final grades of year one, the means of the first three years, the means of years four to six, the cumulative means of the six years, and the final examination grades in the different disciplines.

**Table 1:** Demographics of the study cohorts

Variable		Cohorts 1+2 (2013-2014)		p-value	Cohort 3 (2015)		p-value
		pre-med (N=29)	med (N=167)		pre-med (N=36)	med (N=92)	
Gender N (%)	M	13 (44.8)	69 (41.3)	0.724	8 (22.2)	32 (34.8)	0.168
	F	16 (55.2)	98 (58.7)		28 (77.8)	60 (65.2)	
Age	Mean (SD)	31.76 (2.61)	31.69 (2.10)	0.886	30.14 (1.83)	30.94 (2.02)	^*^0.049

^*^p < 0.05, χ^2^ test used for Gender comparison. T-Test used for Age comparison.

In the first and second cohorts, the final grades of year one of the PREMED students were significantly higher than those of the MED students, taking equivalent exams in their content and level. This outcome may be related to the requirements for transitioning into the medical program and the specific study structure of these two cohorts. In the third cohort, we also found a significant difference in the final grades of year one between the two groups, but here the MED students had the higher grades.

No significant differences were found between the groups, for any of the three cohorts, in grades achieved in the following years, except for the final examination in psychiatry in the third cohort, with the mean grade of the MED group higher (M=81.2, SD=4.5) than that of the PREMED group (M=79.7, SD=2.7) with (t_(__119)_=2.27, p=0.025). The results are presented in Table 2.

### Multivariate and quantile regression analyses

In a multivariate analysis, we tested the association between the study track and the final grades after adjusting for the demographic variables of gender and age. We found no significant interactions with the dependent variables. Data were analyzed by linear and quantile regression, which was used to determine the effect on the dependent variable at different grade quantiles and the effect on the mean grade.

The variable' study track' was significantly associated with year one grades of students in the first and second cohorts when analyzed by linear regression and quantile regression for quantiles 25, 50, and 70. After adjusting for age and gender, students in the PREMED track had higher grades than students in the MED track. In all other years of study and the final examinations, the variable 'study track' was not associated with the grades. In the third cohort, no significant effect was found for any of the study years. However, in the final examinations of the third cohort, the variable 'study track' had a significant effect on the grades in the high quantiles: 75, 90, and 95.

After adjusting for gender and age, students in the PREMED track had lower grades than students in the MED track. The results are presented in Table 3.

**Table 2:** Final grades analysis of students in both MED and PREMED tracks

Year/grade	Cohorts 1+2 (2013-2014)		p-value	Cohort 3 (2015)		p-value
	Pre-med (N=29)	Med (N=167)		Pre-med (N=36)	Pre-med (N=92)	
Year 1 Median (Min- max) Mean (SD)	90.5 (78.9-96.4) 89.91 (4.11)	86.9 (73.9-97.1) 86.73 (4.08)	<0.01^*^	85.3 (74.7-93.0) 85.09 (4.31)	86.5 (78.1-95.1) 86.63 (3.73)	0.047
Mean Year 1-3 Median (Min-max) Mean (SD)	85.4 (76.4-92.9) 85.63 (4.34)	84.2 (75.1-95.3) 84.36 (4.48)	0.159	82.7 (77.1-92.7) 83.17 (3.99)	83.8 (76.2-93.5) 84.21 (3.81)	0.171
Mean Year 4-6 Median (Min-max) Mean (SD)	85.6 (81.4-91.2) 85.86 (2.85)	85.6 (79.5-94.1) 85.82 (2.98)	0.941	85.9 (81.8-93.3) 86.29 (2.70)	86.9 (80.2-93.2) 87.22 (2.79)	0.092
Internal Final exam Median (Min-max) Mean (SD)	85.0 (73-94) 83.76 (5.55)	82.0 (71-95) 82.07 (5.21)	0.112	82.5 (69-92) 82.39 (5.11)	83.5 (74-93) 83.63 (4.52)	0.181
Pediatric Final exam Median (Min-max) Mean (SD)	83.0 (72-89) 82.69 (3.89)	83.0 (74-92) 82.83 (3.57)	0.848	82 (73-90) 82.58 (3.67)	83 (73-92) 83.47 (3.92)	0.242
Surgery Final exam Median (Min-max) Mean (SD)	78.0 (70-87) 78.41 (4.49)	78.0 (66-87) 77.85 (4.31)	0.518	77.5 (69-85) 77.65 (4.25)	77 (59-87) 77.61 (4.33)	0.967
Psychiatry Final exam Median (Min-max) Mean (SD)	84.0 (76-92) 83.45 (4.26)	83.0 (71-92) 82.68 (4.19)	0.367	80 (76-86) 79.74 (2.66)	81.5 (70-92) 81.24 (4.50)	0.025^*^
Gynecology Final exam Median (Min-max) Mean (SD)	81.0 (75-88) 81.45 (3.22)	81.0 (68-94) 81.12 (4.47)	0.709	83 (71-90) 82.74 (4.00)	82 (70-93) 82.17 (4.37)	0.525
Cumulative average Median (Min-max) Mean (SD)	85.2 (80.4-92.2) 85.75 (3.67)	85.0 (78.4-94.8) 84.98 (3.67)	0.30	84.3 (79.5-93.0) 84.42 (3.34)	85.1 (77.6-92.9) 85.39 (3.18)	0.130

^*^p < 0.05, T-Test used for all analyses

**Table 3:** Adjusted final grades of students in both MED and PREMED tracks

Cohort		Linear regression	Quantile Regression						p-value
			q=0.1	q=0.25	q=0.5	q=0.75	q=0.9	q=0.95	
Cohorts 1+2 (2014-2013)	Year 1	3.06^**^	2.74	3.07^**^	2.45^**^	3.60^**^	1.68	1.10	p=0.001
	Year 1-3 (mean)	1.09	0.40	2.00	0.95	2.00	0.51	-0.84	p=0.217
	Year 4-6 (mean)	0.04	0.58	0.36	-0.43	0.37	-0.27	-0.74	p=0.225
	Year 1-6 (mean)	0.64	0.40	1.00	0.55	1.02	0.31	-0.78	p=0.247
	Internal Final exam	1.69	1.00	0.33	2.33	1.50	1.50	0.67	p=0.008
	Pediatric Final exam	-0.31	0.00	0.33	-1.00	-0.83	0.25	-1.00	p=0.171
	Surgery Final exam	0.51	-0.29	0.44	0.33	0.00	1.40	1.67	p=0.223
	Psychiatry Final exam	0.99	0.00	0.67	1.60	1.00	1.00	-0.67	p=0.335
	Gynecology Final exam	0.23	1.57	0.50	0.40	0.00	-1.00	-1.33	p=0.596
Cohort 3 (2015)	Year 1	-1.15	-1.87	-1.40	-0.60	-0.90	0.07	0.07	p=0.073
	Year 1-3 (mean)	-0.98	-1.60	-0.48	-1.10	-1.50	-0.90	-1.22	p=0.334
	Year 4-6 (mean)	-1.25^*^	-0.90	-1.50	-1.30	-1.85	-1.50	-0.57	p=0.103
	Year 1-6 (mean)	-1.06	-1.39^*^	-0.60	-0.70	-1.80	-0.20	-1.00	p=0.332
	Internal Final exam	-2.02^*^	-2.67	-2.57	-2.20	-3.00^*^	0.00	-0.50	p=0.064
	Pediatric Final exam	-1.10	0.00	-0.75	-1.00	-1.50	-1.00	-1.00	p=0.291
	Surgery Final exam	-1.10	0.00	-1.40	0.38	-0.67	1.00	-1.67*	p=0.223
	Psychiatry Final exam	-1.38	2.13	-0.40	-1.00	-4.00^**^	-5.50^**^	-6.00^**^	p=0.335
	Gynecology Final exam	0.34	1.45	0.67	1.17	0.00	-2.00	-1.00	p=0.330

^*^p-value<0.05, ^**^p-value<0.01. Multivariate and quantile regression adjusted for age and gender. PREMED is the dummy variable obtaining value 1 if the student is in PREMED track, and 0 if in the MED track, all values presented as PREMED-MED.

**Table 4:** Comparison of both MED and PREMED tracks students, who chose the MD/PhD track

Student	MD\PHD N (%)	MD N (%)	Total	p-value
201-20138				
Pre-Med	3 (27.3)	8 (72.7)	11	p=0.072
Med	7 (9)	71 (91)	78	
Total	10 (11)	79 (89)	89	
2014-2019				
Pre-Med	2 (11.1)	16 (88.9)	18	p=0.406
Med	5 (5.7)	82 (94.3)	87	
Total	7 (6.7)	98 (93.3)	105	
2015-2020				
Pre-Med	2 (5.6)	34 (94.4)	36	p= 0.564
Med	3 (3.3)	87 (96.7)	90	
Total	5 (4)	121 (96)	126	

p < 0.05, Fisher exact test used for all analyses.

### Association with the MD/PhD track selection

Only 22 students (0.07%) selected the MD/PhD track. In all three cohorts, we noticed that the percentage of students who chose to join the integrated MD/PhD track was higher among PREMED students than the MED track students (201-20138: 27.3% vs 7 (9%); 2014-2019: 2 (11.1%) vs 5 (5.7%); 2015-2020: 2 (5.6%) vs 3 (3.3%)). Yet, we found no significant difference between students in the PREMED or MED track in joining the MD/PhD program, despite the additional focus of the PREMED program by biomedical research course (Table 4).

## Discussion

PREMED programs throughout the world aim to assist in selecting medical students who meet specific cognitive and personality criteria. The selection criteria are based on qualities that would be essential for the students in their future work as physicians. The variability between programs in different schools stems from the particular nature of the local or international medical school, the local or international health needs, and the admission quotas that are appropriate for each medical school worldwide.

In addition, admission scores have been shown to correlate directly with the level of academic success, and academic achievements in PREMED have been shown to correlate with those in preclinical years.^6^^,^^7^^,^^13^ The ability of the different programs to influence the ethical aspects of a student's personality is still controversial, and studies determining the effects of the PREMED program on these aspects remain inconclusive.^10^ In addition, it is known that students' ambition and motivation change during their years of study and throughout their clinical and academic careers.^14^

These differences in motivation are well reflected in the achievements of the three PREMED cohorts of our study. Overall, there was no difference in the academic achievements between students in the PREMED track and those in the MED track. However, the grades of the two first cohorts of the PREMED students, whose transition to the medical program was based on academic achievements, were significantly higher than the grades of students in the third cohort and of students in the direct track. Several factors were likely to contribute to this increased motivation of PREMED students in the first two cohorts. These include the fact that the separate PREMED admission track offered a unique opportunity for students who would not have been accepted to the medical school in previous years and the fact that only a proportion of the PREMED students were able to join the medical program. The finding of the higher achievements of PREMED students is consistent with the literature but was unique here, given the exceptional circumstances of our program. Evidently, students in the third cohort, who were directly integrated into the medical program, were less ambitious than students in the two previous cohorts.

Further evidence for the effect of motivation on academic achievements was provided by the results of the two first PREMED cohorts in the later years of study. Once the students were notified that they had been accepted into the medical program, their mean grades decreased and were no longer different from those of the other students. The acceptance to the medical program apparently reduced the pressure and somewhat decreased grades. However, according to the quantile analysis, students who were highly motivated from the beginning maintained their level of motivation. This is demonstrated by the observation that students with high grades had even higher grades than those in the direct MED track in later years. Students of the first PREMED cohort maintained top grades throughout their years in academia, and perhaps this is a result of selection bias or a higher level of commitment. Our study also found that PREMED students in all cohorts were more motivated to join the MD/PhD research track, but this was not significant because of the small number of participants.

The PREMED opportunity that was provided to only three cohorts raises a disturbing, practical concern about all those students rejected from medical studies because of the limited number of available places. The achievements of the three consecutive student cohorts over the years of study and upon the completion of their studies suggest there is no difference between candidates in places 1 to 100 and 150 to 400 in the ranking of the admissions committee. Thus, the admissions committee does not predict academic achievements, at least not for places 1-400. It appears that in each yearly intake, we are missing out on 150-300 potential doctors that could have achieved well academically had they been trained in Israel, although we cannot be certain about their inter-personal capabilities, as they had been rejected by the admissions committee. This figure is consistent with the literature, which shows that the PREMED students achieve similar results to graduates in the direct medical programs.^15^ This information evokes ethical and social questions regarding the mechanisms for admission, including the PREMED programs and their implications on the medical profession. Many countries are losing good and worthy candidates, who would have likely graduated from medical school as successful as their peers, but who missed out on this opportunity.^16^

Alternatively, if we revised the admission scheme and accepted all medical candidates through a PREMED program, we could have had a yearly intake of 400 students. However, because placements for clinical training would still be limited, it would be necessary to employ some form of selection at the end of the three years and before admission to the clinical years. Academic grades by themselves appear to be an inadequate selection tool, and therefore, interviews and personality tests will still be required. Eventually, the same group of candidates might miss out on medical training, and this proposed admission scheme would have no advantage over the existing one. Furthermore, this lengthy process might discourage candidates who would not be motivated to join such a program.

At the same time, the Ministry of Health and the Council for Higher Education in Israel intend to continue increasing Israeli medical students' annual intake from 750 to 1000 in the coming years for two main reasons. The first is a shortage of physicians per capita, which could be addressed by promoting relatively fast training tracks for physicians in Israel and abroad. The second is the need to train physicians with a good education adapted to the Israeli health system.

In practice, there has been a significant increase in the number of Israeli students studying medicine abroad. Currently, approximately six out of ten new doctors in Israel have been trained and qualified abroad - a proportion that is the highest in the OECD countries. The implications of medical training abroad are twofold: the theoretical and professional training is sometimes provided by institutions known to have a substandard level of teaching, and there is no real opportunity for educational influence on the character of a medical doctor who returns to the country already qualified.

Considering these challenges, forums on medical education often debate whether the time has come to change the model of medical studies.^16^^,^^17^ On the one hand, the six-year programs offer the deans an extended period and more diverse means to shape and influence the students. On the other hand, four-year programs have the advantage of accepting mature students from diverse academic backgrounds, including physics, nursing, East Asian studies, philosophy, and chemistry. Mature students admitted to medical schools can choose various tracks from the start, such as conjoint research, entrepreneurship, or management tracks, according to their areas of interest and backgrounds.

We later found that the rates of applicants for the combined MD / PHD track were higher among PREMED track than the rates of applicants in the regular MED class. Although due to the small numbers, no statistical significance was found, there was some positive effect on students obtained in the PREMED track. On the one hand, this could be linked to the essential difference between the two tracks, represented by the research course implemented in PREMED. On the other hand, the third PREMED cohort eventually did not participate in the different learning track at all and yet admitted more than MED students to the combined MD/ PHD. We may assume that this is an example of the ability to influence the character design of learners by diversifying the medical admission paths.

### Limitations

Our study has several limitations. First, the PREMED track that we trialed as a pilot program for three cohorts was basically different from PREMED programs in other schools throughout the world. Second, the program was originally intended as an alternative pathway into medical studies, enhanced by a research component. The program was achievement-based and designed for eligible candidates who met the threshold criteria but were not admitted to medical studies because of the limited number of places. Eventually, the program was not fully executed, and the changes introduced each year practically abolished it. However, we thought it was worthwhile to present the results of this pilot program. Now that the graduates of the three cohorts have completed their medical studies, a retrospective examination of their academic achievements can be used to learn about medical school candidates, and particularly those who are not accepted into the medical program. The study also allows us to evaluate the wider implications of the admission processes.

## Conclusions

In conclusion, our study demonstrated no significant difference in association between the achievements of medical school graduates and their admission track. We have also shown the importance of the PREMED alternative track in reducing the loss of worthy candidates. We believe that these results, which are consistent with international findings, should provoke further discussion and consideration of the significant potential loss of human resources. Moreover, considering reinstatement of PREMED programs will allow candidates who are determined to study medicine and meet the required academic threshold to prove themselves, despite them being rejected by the admissions committee. Admission to the PREMED program would still depend on interviews and personality tests, which would exclude candidates who present with substantial personality barriers that are incompatible with the image of a medical doctor. It appears that additional admission tracks could provide creative admission alternatives, such as a community-oriented track, a research-oriented track, and others. The additional tracks would be invaluable in maintaining eligible candidates and influencing their education and training in local medical schools. Furthermore, additional admission tracks could potentially retain candidates who would otherwise give up on their medical aspirations and choose a different career. Further research is needed in search of diverse ways to successfully implement PREMED programs, and to face the challenge of successful medical student selection while reducing the loss of many worthy potential physicians.

### Conflict of Interest

The authors declare that they have no conflict of interest.
